# Evaluating the Power of GPU Acceleration for IDW Interpolation Algorithm

**DOI:** 10.1155/2014/171574

**Published:** 2014-02-23

**Authors:** Gang Mei

**Affiliations:** Institute of Earth and Environmental Science, University of Freiburg, Albertstraße 23B, 79104 Freiburg im Breisgau, Germany

## Abstract

We first present two GPU implementations of the standard Inverse Distance Weighting (IDW) interpolation algorithm, the tiled version that takes advantage of shared memory and the CDP version that is implemented using CUDA Dynamic Parallelism (CDP). Then we evaluate the power of GPU acceleration for IDW interpolation algorithm by comparing the performance of CPU implementation with three GPU implementations, that is, the naive version, the tiled version, and the CDP version. Experimental results show that the tilted version has the speedups of 120x and 670x over the CPU version when the power parameter *p* is set to 2 and 3.0, respectively. In addition, compared to the naive GPU implementation, the tiled version is about two times faster. However, the CDP version is 4.8x∼6.0x slower than the naive GPU version, and therefore does not have any potential advantages in practical applications.

## 1. Introduction

Spatial interpolation is a fundamental task in Geosciences, where a number of data points with some kinds of known values such as elevations are used to predict unknown quantities of a continuous phenomenon for the prediction points. The computational cost of the underlying algorithms usually grows with the number of data entering the interpolation and the number of locations for which interpolated values are needed. Typically, the implementation of spatial interpolation within the conventional sequential programming patterns is computationally expensive for a large number of data sets and thus calls for scalable computing solutions such as parallelization.

The Inverse Distance Weighting (IDW) algorithm is one of the most commonly used spatial interpolation methods in Geosciences mainly due to its straightforward implementation. Shepard [1] proposed a two-dimensional interpolation function for irregularly spaced data; this function is the basis for IDW-based interpolations. In the basic form of the Shepard's method, all data points are used to calculate the interpolated values. In order to reduce computational cost, some efficient implementations of the IDW interpolation have been carried out in various massively parallel computing environments on multicore CPUs and/or GPUs platforms.

By taking advantage of the power of traditional CPU-based parallel programming models, Armstrong and Marciano [2, 3] implemented the IDW interpolation algorithm in parallel using FORTRAN 77 on shared-memory parallel supercomputers and achieved an efficiency close to 0.9. Guan and Wu [4] performed their parallel IDW algorithms and used open multiprocessing (OpenMP) running on an Intel Xeon 5310, achieving an excellent efficiency of 0.92. Huang et al. [5] designed a parallel IDW interpolation algorithm with message passing interface (MPI) by incorporating single process, multiple data (SPMD), and master/slave (M/S) programming modes and attained a speedup factor of almost 6 and an efficiency greater than 0.93 under a Linux cluster linked with six independent PCs.

Since that general purpose computing on modern Graphics Processor Units (GPUs) can significantly reduce computational cost by performing massively parallel computing, current research efforts are being devoted to parallel IDW algorithms on GPU computing architectures such as CUDA [6] and OpenCL [7]. For example, Huraj et al. [8, 9] have deployed IDW on GPUs to accelerate snow cover depth prediction. Henneböhl et al. [10] studied the behavior of IDW on a single GPU depending on the number of data values, the number of prediction locations, and different ratios of data size and prediction locations. Hanzer [11] implemented the standard IDW algorithm using Thrust, PGI Accelerator, and OpenCL. Xia et al. [12, 13] developed the GPU implementations of an optimized IDW algorithm proposed by them and obtained 13- to 33-fold speedups in computational time over the sequential version.

When intending to profit from massively parallel computing on GPUs, algorithms are needed to be carefully implemented according to the inherent features of GPU computing architectures. For example, shared memory is expected to be much faster than global memory; thus any opportunity to replace global memory access by shared memory access should therefore be exploited [6].

In this paper, we first develop a GPU implementation of the IDW algorithm according to the strategy “tiling” [14] for reducing global memory access by taking advantages of shared memory. This implementation is called the *tiled* version. We also perform the implementation of IDW interpolation algorithm by exploring one of the latest features of CUDA architecture, the Dynamic Parallelism (CDP) on a single GPU. The feature CDP allows nested levels of parallelism and thus is highly suitable to be used to implement the IDW algorithm. We finally carry out several experimental tests to evaluate the performance of our tiled version and CDP version when compared to the CPU version and the naive GPU version.

The paper is organized as follows.  Section 2 gives a brief introduction to the CUDA architecture and Dynamic Parallelism and the principle of the IDW algorithm.  Section 3 concentrates mainly on our GPU implementations of the standard IDW algorithm using the strategy “tiling” and the feature CUDA Dynamic Parallelism.  Section 4 presents several experimental tests and discusses the results. Finally, Section 5 draws some conclusions.

## 2. Backgrounds

### 2.1. CUDA and Dynamic Parallelism

CUDA (Compute Unified Device Architecture) is a general purpose parallel computing platform and programming model created by NVIDIA and implemented by the NVIDIA GPUs, which leverages the power of parallel computing on GPUs to solve complex computational problems in a more efficient way than on a CPU. CUDA comes with a software environment that allows developers to use C as a high level programming language. More details of the CUDA architecture are presented in [6].

**Figure 1:**
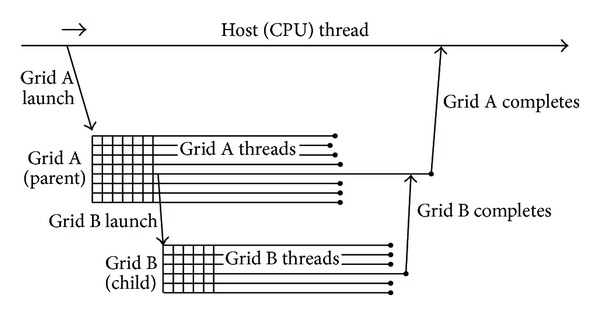
Parent-child launch nesting (derived from Figure 12 in [6]).

Dynamic Parallelism in CUDA is an extension to the CUDA programming model that enables a CUDA kernel to create and synchronize new nested work by launching nested kernels [6]; see Figure 1. In those CUDA systems that do not support the Dynamic Parallelism, multiple kernels can only be launched from the host code in sequence. However, the Dynamic Parallelism allows CUDA kernels to dynamically launch other kernels without burdening the host.

Dynamic Parallelism introduces the concepts of “parent” and “child” grids. A parent grid is one that has launched new nested grid(s), that is, the child grid(s). A child grid is one that has been launched by a parent grid. A child grid must be complete before its parent grid is considered complete; in other words, the parent is not considered complete until all of its launched child grids have also been completed [6].

There are several implementation limitations when programming the Dynamic Parallelism. For example, global memory, constant memory, and texture memory are visible for both the parent and child grids and can be written within the parent and child grids coherently. However, the operations of above three types of memories in the parent thread prior to the child thread's invocation are visible to the child grid; all memory operations of the child grid are visible to the parent after the parent has synchronized on the child grid's competition. Shared memory and local memory are private storage for a thread block or a thread, respectively, which are not visible outside their scopes. It is *illegal* to pass a pointer to shared memory or local memory as a launch argument when launching a child kernel.

Dynamic Parallelism is introduced with the Kepler architecture that has the Compute Capability 3.5 or higher.

### 2.2. IDW Interpolation

The IDW algorithm is one of the most commonly used spatial interpolation methods in Geosciences, which calculates the interpolated values of unknown points (prediction points) by weighting average of the values of known points (data points). The name given to this type of methods was motivated by the weighted average applied since it resorts to the inverse of the distance to each known point when calculating the weights. The difference between different forms of IDW interpolation is that they calculate the weights variously.

A general form of predicting an interpolated value *Z* at a given point *x* based on samples *Z*_*i*_ = *Z*(*x*_*i*_) for *i* = 1, 2, …, *n* using IDW is an interpolating function is

(1) Z(x)=∑i=1nωi(x)zi∑j=1nωj(x),  ωi(x)=1d(x,xi)p.

The above equation is a simple IDW weighting function, as defined by Shepard [1], where *x* denotes a predication location, *x*_*i*_ is a data point, *d* is the distance from the known data point *x*_*i*_ to the unknown prediction point *x*, *n* is the total number of data points used in interpolating, and *p* is an arbitrary positive real number called the power parameter (typically, *p* = 2).

## 3. GPU Implementations

### 3.1. The Naive Version

The naive implementation of the IDW interpolation is straightforward. Assuming that there are *m* data points used to evaluate the interpolated values for *n* prediction points, each thread within a grid is invoked to calculate the distances to all data points, the inverse weights, and the weighted average (i.e., the interpolated value) of one predication point. Obviously, it is needed to allocate *n* threads within a thread grid.

The implemented CUDA kernel of this naive version can be found in [8]. In this naive implementation, only registers and global memory are used without profiting from the use of shared memory.

### 3.2. The Tiled Version

The CUDA kernel presented in [8] is a straightforward implementation of IDW algorithm that does not take advantage of shared memory. Each thread needs to read the coordinates of all data point from global memory. Thus, the coordinates of all data points are needed to be read *n* times, where *n* is the number of predication points.

In CUDA architecture, shared memory is expected to be much faster than global memory; thus, any opportunity to replace global memory access by shared memory access should therefore be exploited [6]. A common optimization strategy is called “tiling,” which partitions the data stored in global memory into subsets called *tiles* so that each tile fits into the shared memory [14].

This optimization strategy “tiling” is adopted to implement the IDW interpolation: the coordinates of data points is first transferred from global memory to shared memory; then each thread within a thread block can access the coordinates stored in shared memory concurrently. Since shared memory is limited per SM (Stream Multiprocessor), the data in global memory, that is, the coordinates of data points, needs to be first split/tiled into small pieces and then transferred to shared memory.

In the tiled implementation, the tile size is set as the same as the block size (i.e., the number of threads per block). Each thread within a thread block is responsible for loading the coordinates of one data point from global memory to shared memory and then computing the distances and inverse weights to those data points stored in current shared memory. After all threads within a block finished computing these partial distances and weights, next piece of data in global memory is loaded to shared memory and used to calculate current wave of partial distances and weights. Each thread accumulates the results of all partial weights and all weighted values into two registers. Finally, the interpolated value of each prediction point can be obtained according to the sums of all partial weights and weighted values and then written into global memory.

By blocking the computation this way, the access to global memory can be reduced since the coordinates of data points are only read (*n*/threadsPerBlock) times rather than *n* times from global memory, where *n* is the number of predication points and threadsPerBlock denotes the number of threads per block.

### 3.3. The CDP Version

The basic idea behind implementing the IDW interpolation using Dynamic Parallelism is as follows. There are two levels of parallelism in IDW interpolation.

1. Level 1: for all prediction points, the interpolated values can be calculated in parallel. The interpolating for each unknown point does not depend on that of any of other points and thus can be carried out concurrently.
2. Level 2: for each prediction point, it is needed to first calculate the distances to all data points and then the inverse weights. These distances and weights can obviously be calculated in parallel.

The parent kernel is responsible for performing the first level of parallelism, while the child kernel takes responsibility for realizing the second level of parallelism. There are only two levels of kernel launches.

In more details, the parent grid is responsible for calculating the interpolated values of all prediction points in parallel. Each thread within the parent grid is designed to evaluate the interpolated value of one prediction point by invoking the child grid. Hence, there are at least *n* threads created in the parent grid.

The launch arguments of the parent kernel mainly include the coordinates of data points and prediction points. Several arrays are allocated in global memory to store these coordinates. In addition, another array, sum [*n*], is needed to be allocated to store the intermediate value, that is, the accumulation of *m* inverse weights, where *m* is the number of all data points. Similarly, the array *pz*[*n*], which is originally allocated to store the final interpolated values of all predication points, is temporarily used to store the accumulation of the weighted values. Therefore, the final interpolated value of the *i*th prediction point is the division of *pz*[*i*] and sum[*i*], that is, *pz*[*i*] = *pz*[*i*]/sum [*i*].

In the child grid, each thread within a block is responsible for computing the distance of one data point to the prediction point specified by the parent thread. The distance is first stored as a register in each thread, and then transferred to the shared memory. After finishing computing all distances, the parallel reduction introduced by Harris [15] is carried out within each thread block to obtain the partial accumulations of the inversed distances and the weighted values. Finally, all partial accumulations that are temporarily stored as two registers are wrote back to global memory.

The arguments of the child kernel are almost the same as those of the parent kernel. The only difference is that there is an additional argument, the unique index of the thread in the parent grid. This argument is used to indicate which thread in the parent grid invokes the child kernel.

## 4. Results and Discussion

### 4.1. Experimental Results

We evaluate the GPU implementations using the NVIDIA GeForce GT640 (GDDR5) graphics cards with CUDA 5.5. Note that the GeForce GT640 card with memory GDDR5 has the Compute Capability (CC) 3.5, while it only has Compute Capability 2.1 with the memory DDR3. The CPU experiments were performed on Windows 7 SP1 with a dual Intel i5 3.2 GHz processor and 8 GB of RAM memory. For each set of the testing data, we carry out one CPU implementation and three GPU implementations only on single precision.

For each implementation, we perform two different forms that have different values of the power parameter *p*: in the first form, the power *p*, see (1), is set to an integer value 2, while this value is set to 3.0 in the second form.

Different specifications of the power *p* lead to different designs of each implementation: when *p* is set to 2, the distance from the *i*th prediction point to the *j*th data point can be simply obtained via the formulation, dis = (*px*[*i*] − *dx*[*j*])∗(*px*[*i*] − *dx*[*j*])+(*py*[*i*]−*dy*[*j*])∗(*py*[*i*] − *dy*[*j*]), where *px* and *py* and *dx* and *dy* are the coordinates of prediction points and data points; according to above formulation, it is not needed to calculate the square root of the value dis. In contrast, when the power *p* is given as 3.0, it is needed to first calculate the square root (i.e., the distance) and then the powered value dis^*p*^. In practical implementations, in order to avoid calculating square roots, we use the following formulation to calculate the powered distance, dis^*p*^ = powf(dis, 0.5∗*p*).

The input of the IDW interpolation is obviously the coordinates of the data points and prediction points. The performance of the CPU and GPU implementations may differ due to different sizes of input data [10, 11]. Hence, we perform the tests in three cases in terms of the numbers of the data points and prediction points as follows:

1. the numbers of prediction points and data points are identical;
2. the number of data points is fixed, and the number of prediction points differs;
3. the number of prediction points is fixed, and the number of data points differs.

**Figure 2:**
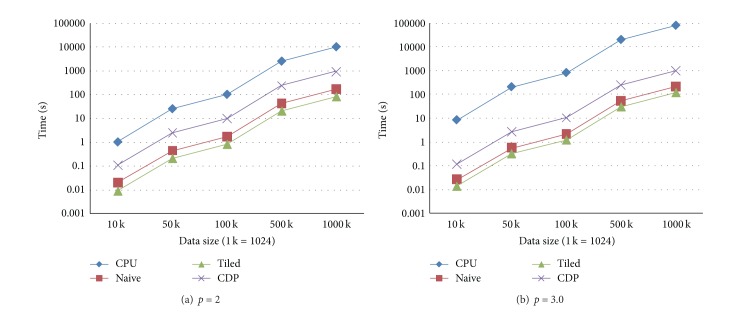
Execution times in the test case when the numbers of prediction points and data points are identical. (a) The form in which the power parameter is set to 2. (b) The form in which the power parameter is set to 3.0.

**Figure 3:**
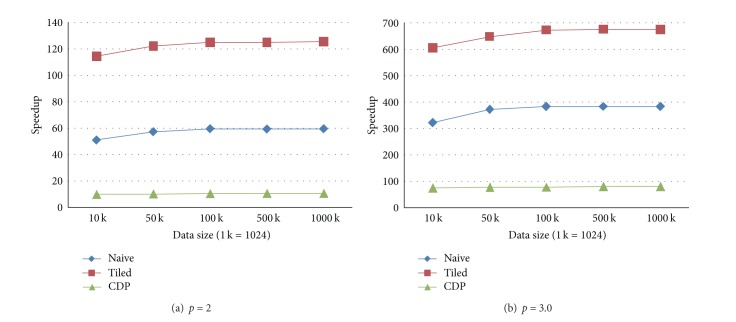
Speedups in the test case when the numbers of prediction points and data points are identical. (a) The form in which the power parameter is set to 2. (b) The form in which the power parameter is set to 3.0.

We create five groups of sizes, that is, 10 K, 50 K, 100 K, 500 K, and 1000 K (1 K = 1024). When the number of prediction points is identical to the number of data points, five tests are performed by setting the numbers of both the data points and prediction points as the above listed five groups of sizes. The execution times of four implementations and corresponding speedups are shown in Figures 2 and 3, respectively.

**Figure 4:**
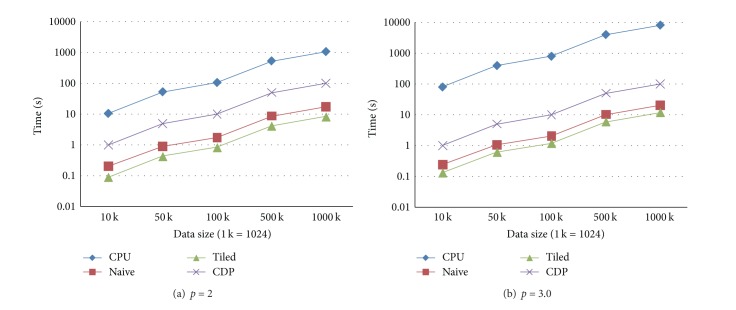
Execution times in the test case when the number of data points is fixed and the number of prediction points differs. (a) The form in which the power parameter is set to 2. (b) The form in which the power parameter is set to 3.0.

**Figure 5:**
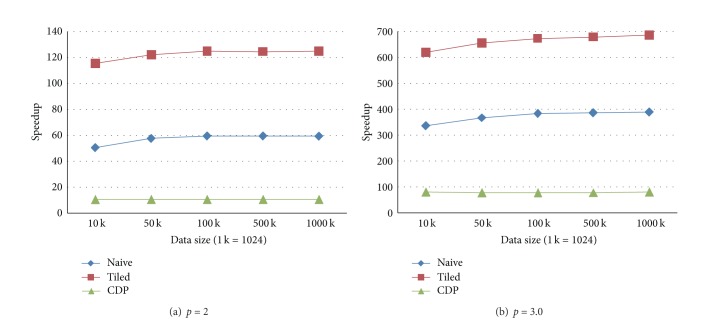
Speedups in the test case when the number of data points is fixed and the number of prediction points differs. (a) The form in which the power parameter is set to 2. (b) The form in which the power parameter is set to 3.0.

In the second test case, the number of data points is fixed as 100 K. Five tests are also carried out by setting the sizes of the prediction points as the above listed five groups of size. The experimental results in this case are presented in Figures 4 and 5.

**Figure 6:**
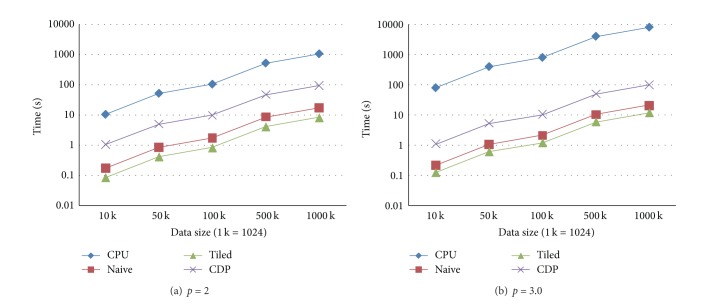
Execution times in the test case when the number of prediction points is fixed, and the number of data points differs. (a) The form in which the power parameter is set to 2. (b) The form in which the power parameter is set to 3.0.

**Figure 7:**
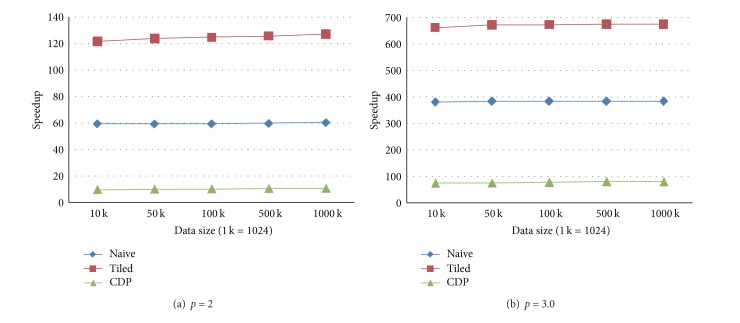
Speedups in the test case when the number of prediction points is fixed, and the number of data points differs. (a) The form in which the power parameter is set to 2. (b) The form in which the power parameter is set to 3.0.

Different from the second test case, in the third case, the number of the prediction points rather than the data points is fixed as 100 K. And the number of data points is set to one of the five groups of size. The results of five experimental tests are shown in Figures 6 and 7.

According to the results generated in above three test cases, we have found that when the power is set to 2, the GPU implementations, that is, the naive version, the tiled version, and the CDP version achieve the speedups of 60x, 120x, and 10x over the CPU implementation, respectively; in contrast, when the power is set to 3.0, the speedups are about 380x, 670x, and 78x for the naive, the title, and the CDP implementations, respectively.

### 4.2. Discussion

Several GPU implementations of the IDW interpolation are presented in the literature [8, 10–12]. And the comparable results of those GPU implementations over corresponding CPU ones are also reported. For example, Xia et al. [12] implemented their optimized IDW algorithm and obtained 13- to 33-fold speedups in computation time over the sequential version. Hanzer [11] implemented the standard IDW algorithm using different approaches (Thrust, PGI Accelerator, and OpenCL) and achieved a peak speedup of 140x.

In this paper, we develop two GPU implementations of IDW interpolation, the tiled version and the CDP version, by taking advantage of the fast shared memory and the CUDA Dynamic Parallelism. To the best of our knowledge, the above two GPU implementations have not been introduced in existing literatures.

In the tiled version, the coordinates of data points originally stored in global memory are divided into small pieces/tiles that fit the size of shared memory and then loaded from slow global memory to fast shared memory. These coordinates stored in shared memory can be accessed quite fast by all threads within a thread block when calculating the distances. By blocking the computation this way, we take advantage of fast shared memory and reduce the global memory access: the coordinates of data points are only read (*n*/threadsPerBlock) times from global memory, where *n* is the number of prediction points.

Experimental tests show that the tilted version has the speedups of 120x and 670x over the CPU version when the power parameter *p* is set to 2 and 3.0, respectively. In addition, compared to the naive GPU version, the tiled implementation is about two times faster.

The basic idea behind implementing the IDW interpolation using Dynamic Parallelism is simple. There are two levels of parallelism in IDW interpolation: (1) level 1: for all prediction points, the interpolated values can be calculated in parallel; (2) level 2: for each prediction point, the distances to all data points can be calculated in parallel. The parent kernel is responsible for performing the first level of parallelism, while the child kernel takes responsibility for realizing the second level of parallelism.

In the standard IDW interpolation, it needs to calculate *m*∗*n* distances and corresponding weights when there are *m* data points and *n* prediction points. The two levels of parallelism in the CDP version theoretically allows the IDW interpolation operator to calculate all of these *m*∗*n* distances in parallel and thus reduce the computational cost.

However, we obtain a negative result in practice. Although the CDP version is about 10x and 78x times faster than the CPU version when the power *p* is set to 2 and 3.0, respectively, it is 4.8x~6.0x slower than the naive GPU version. Therefore, the CDP version does not have any potential advantages in practical applications.

We analyze the CDP implementation carefully to explain the negative behavior and find that there are probably two main causes.

(*1)   No Optimization in the Use of Global Memory.* When programming Dynamic Parallelism, global memory, constant memory, and texture memory are visible for both the parent and child grids and can be written within the parent and child grids coherently. Shared memory and local memory are private storage for a thread block or a thread, respectively, which are not visible outside their scopes. It is illegal to pass a pointer to shared memory or local memory as a launch argument when launching a child kernel.

In the CDP version, the input data are the coordinates of data points and prediction points, which is originally stored in global memory. When a thread within the child grid is invoked to calculate the distances of one prediction point to all data points, only those coordinates stored in global memory can be passed as a launch argument from the parent kernel to the child kernel. The “tiling” optimization strategy described in the tiled version cannot be accepted to reduce the global memory access since the coordinates that are first divided and then loaded to shared memory cannot be passed as a launch argument when launching a child kernel.

Due to above implementation limitations of Dynamic Parallelism, there are currently no optimization approaches to reducing global memory access. The coordinates of all data points that are stored in global memory are needed to be read n times, where n is the number of prediction points. The amount of global memory access in the CDP version is the same as that in the naive GPU implementation and greater than that in the tiled GPU implementation. This is one of the main causes that lead to the negative result.

*(2)   The Use of the Barrier cudaDeviceSynchronize().* “The cudaDeviceSynchronize() function will synchronize on all work launched by any thread in the thread-block up to the point where cudaDeviceSynchronize() was called. When a parent thread block launches a child grid, the child is not guaranteed to begin execution until the parent thread block reaches an explicit synchronization point (e.g., the calling of cudaDeviceSynchronize()).” [1].

**Figure 8:**
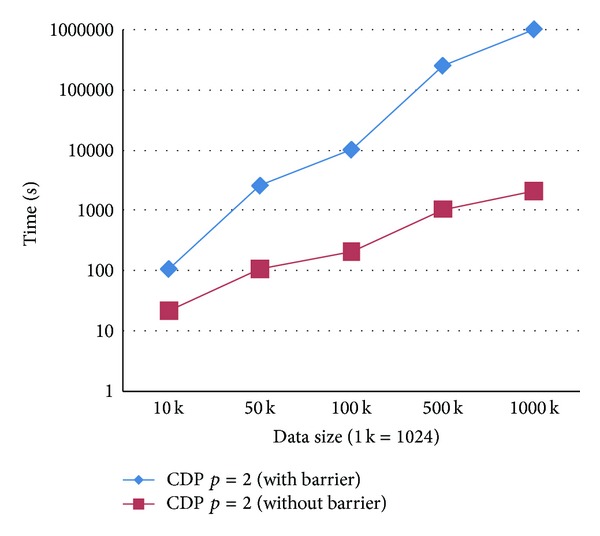
Impact of the barrier cudaDeviceSynchronize() on execution times.

In CDP version, we call the function cudaDeviceSynchronize() after launching the child kernel to guarantee all child grids completely executed. We have observed that, without calling the barrier cudaDeviceSynchronize(), only part of the threads within the parent kernel execute and return expected interpolated values; in other words, the interpolation results in this case are incorrect and uncompleted. However, the execution time for the overall interpolation procedure is much less than that when calling the barrier; see Figure 8.

As noted above, the barrier cudaDeviceSynchronize() is needed to guarantee producing correct and complete interpolating results but is time consuming. This is probably another cause that makes the CDP implementation computationally expensive.

For both the CPU and GPU implementations, the form that has the power set to 3.0 is more computationally expensive than the form where the power is set to 2. In particular, this behavior can be clearly observed for the CPU implementation: the form with the power set to 3.0 is 7.6x slower than the other form. The above behavior is due to the expensive sequential calculations of the powered distances. For those GPU implementations, the deceleration is slight because of the massively parallel computation of the powered distances.

In this paper, both the tiled version and the CDP version are the GPU implementations of the basic form of IDW interpolation algorithm, which calculate the interpolated values using all data points (sample points). A practical solution to reducing the computational cost is to use part of rather than all data points to calculate the interpolated values. The selecting of proper partial sets of data points can be carried out by domain decomposition [12] and local searching schemes such as searching the nearest neighbors [16]. Future work should therefore include the implementation and evaluation of those modified IDW interpolation algorithms by taking advantages of the optimization strategy “tiling” and the feature CUDA Dynamic Parallelism.

## 5. Conclusions

We have developed two GPU implementations of the IDW interpolation algorithm, the tiled version and the CDP version, by taking advantage of shared memory and CUDA Dynamic Parallelism. We have demonstrated that the tilted version has the speedups of 120x and 670x over the CPU version when the power parameter *p* is set to 2 and 3.0, respectively. In addition, compared to the naive GPU version, the tiled implementation is about two times faster. We also find that, although the CDP version is about 10x and 78x times faster than the CPU version when the power *p* is set to 2 and 3.0, respectively, it is 4.8x~6.0x slower than the naive GPU version. Therefore, the CDP version does not have any potential advantages in practical applications. It would be interesting to know the causes of the negative performance of the CDP version and apply the CUDA Parallelism Dynamic to the modified IDW interpolation or other spatial interpolation algorithms.
